# Child Murder

**Published:** 1877-07

**Authors:** 


### CHILD MURDER.

Of all the men in this wide world, book-men most lack common sense in their practices. We lived a year of misery in a minute, as walking in glorious old Boston Common, a gentleman remarked that his son had been going to school nearly a year, and that he expected to get the *gold medal* which was awarded to that boy who, for one whole year, had not committed a single fault. Just think for a moment of the intensity of that bondage of hope and fear, of solicitude, of strife by day and night, awake or in dreams, which must have tortured that poor boy's heart, intensified every hour, as the year drew nearer to a close. We really felt as if that teacher ought to have been hung up by the heels, and well scored with apple-tree switches. We never heard his name, and are glad of it, for we should have consigned it to infamy.

